# Hospital-Based Rehabilitation of Patients Who Had Undergone an Open Reduction and Internal Fixation of the Ribs Due to a Flail Chest: Case Series

**DOI:** 10.3390/ijerph192316026

**Published:** 2022-11-30

**Authors:** Nehama Milson, Iuly Treger, Michal Vered, Asaf Acker, Leonid Kalichman

**Affiliations:** 1 Rehabilitation Department, Soroka University Medical Center, Beer Sheva 84101, Israel; 2 Department of Medicine, Faculty for Health Sciences, Ben-Gurion University of the Negev, Beer Sheva 84101, Israel; 3 Department of Physical Therapy, Recanati School for Community Health Professions, Faculty of Health Sciences, Ben-Gurion University of the Negev, Beer Sheva 84105, Israel

**Keywords:** flail chest, flail chest rehabilitation, flail chest surgery, rehabilitation of ribs fracture, ribs fracture

## Abstract

Flail chest, a severe chest injury, is caused by multiple rib fractures. The open reduction and internal fixation (ORIF) of rib fractures is an effective treatment; however, the patients’ subsequent condition remains unsatisfactory in terms of the activities of daily living (ADL) and pain. No research study has, as yet, reported on hospital-based rehabilitation of patients who had undergone an ORIF. Our aim was to evaluate the efficacy of hospital-based rehabilitation of flail chest post-ORIF patients. Physical therapists assessed the pain, functional independence measure (FIM), and the Berg balance test. A total of three females and four males (mean age 59.43 ± 18.88) were hospitalized. A significant reduction in pain was observed (7.00 ± 1.83 upon admission to 4.10 ± 2.05 pre-discharge (Z = −2.07, *p* = 0.027). A significant improvement in FIM (69.43 ± 14.86 upon admission to 113.57 ± 6.40 pre-discharge, Z = −2.37, *p* = 0.018), and the Berg balance test (35.23 ± 5.87 upon admission to 49.50 ± 3.40 pre-discharge, Z = −2.37, *p* = 0.018), was observed. Upon admission, all the patients required moderate to complete ADL assistance. Upon discharge, all were independent for all ADL functions. Patients after flail chest post-ORIF can benefit from hospital-based rehabilitation.

## 1. Introduction

Flail chest, a severe injury, occurs when part of the chest separates from the rest of the chest due to multiple rib fractures, and usually transpires due to a severe blunt injury such as a fall, aggressive cardiopulmonary resuscitation, or a car accident. This injury changes the mobility of the chest, causes respiratory distress, and can contribute to respiratory failure [1]. According to the American Association for the Surgery of Trauma, chest trauma occurs in 20% of major trauma cases and is responsible for 25% of traumatic deaths. Flail chest occurs in about 7% of chest trauma and patients usually require hospitalization. Flail chest occurs in isolation in less than 40% of cases and more often is accompanied by pulmonary contusions, hemo- and pneumo-thorax, head injury, and occasionally major vascular injury. The mortality of flail chest ranges from 10% to 20% but is often due to accompanying injury rather than the flail chest alone. Morbidity is high due to long hospital stays and complicated recovery [2]. Patients with a flail chest are at risk of complications, i.e., rupture of the spleen, liver, myocardium, and lungs, with a mortality rate of up to 33% [3]. Flagel et al. [4] reported a 10% mortality amongst patients with >4 rib fractures. In patients with eight or more fractures, the probability of death increases to 34%. Depending on the severity of the trauma and complications, 23% to 75% of patients with chest injuries require intubation and mechanical ventilation, which is quite challenging due to the difficulty of balancing adequate ventilation and preventing further lung damage [5]. In the past, the treatment of rib fractures consisted of pain management, early immobilization, gradual remobilization, prevention, and treatment of respiratory complications, including mechanical ventilation [6]. Due to intense pain, patients with a flail chest avoid active chest movements, thus, decreasing ventilation and increasing the risk of respiratory complications. Moreover, due to the avoidance of shoulder girdle movements that increase pain, shoulder joint contractures develop, and a decrease in the range of motion (ROM) of the scapula may occur.

For years, physicians have been searching for a surgical technique for a reliable fixation of rib fractures, which would avoid the listed complications. A technique using K-shaped wires with a figure eight has been suggested [7]. The mortality of patients with a flail chest is significantly reduced when an open reduction internal fixation (ORIF) procedure is performed [8]. Surgical stabilization aspires to ensure the stability of the damaged segments of the chest wall in order to shorten mechanical ventilation time, reduce the complications that are associated with the ventilation and deformity, shorten hospitalization, duration of pain, and mortality [9,10,11]. Nevertheless, the patient’s condition after this operation remains unsatisfactory in terms of activities of daily living (ADL), dependence, and limitation of upper limb mobility due to pain in the chest wall. No research study has, as yet, reported on hospital-based rehabilitation of patients undergoing an ORIF procedure due to a flail chest. Therefore, our aim was to evaluate the feasibility and efficacy of hospital-based rehabilitation of patients who had undergone an ORIF procedure due to a flail chest.

## 2. Materials and Methods

### 2.1. Design

Case series.

### 2.2. Setting

Department of Rehabilitation, Soroka University Medical Center, Beer Sheva, Israel.

### 2.3. Sample

Post-ORIF flail chest patients hospitalized in a rehabilitation department from 2018 to 2020. All the patients underwent ORIF in the orthopedic department of Soroka University Medical Center and, when they were clinically stable, were transferred to the rehabilitation department. On average, the patients were transferred to the rehabilitation department 17.0 ± 11.9 days after the trauma. 

### 2.4. Rehabilitation Protocol

All patients received treatment according to the protocol that was developed by the department staff: (1) training of basic functions, i.e., bed functions, bed-wheelchair transfer, and transfer from a supine position to sitting on the edge of the bed; (2) training of various functions, i.e., walking, stair climbing, and descending, higher functions that require dynamic and static balance training; (3) specific muscle exercises (i.e., shoulder girdle and scapula muscles); (4) core muscle exercises; (5) exercises to improve ROM of the upper extremities; (6) pain management by medications and physical therapy (mobilizations, deep tissue massage, scar massage, TENS, etc.); and (7) respiratory physical therapy utilizing the TriFlo Incentive Spirometer, chest mobilization, and heart-lung endurance training. The multidisciplinary program of intensive rehabilitation (five days a week) included three hours of physical therapy, occupational therapy, speech therapy, and psychological intervention. Physical therapy and individual exercises were the main part of the program and lasted about 1.5 h a day. 

### 2.5. Data Collection

Data on the patient’s age, sex, time of the operation, length of hospitalization in the rehabilitation department, pain dynamic determined by the visual analog scale (VAS), functional state dynamic of the functional independence measure (FIM), and the need to use auxiliary aids, were assessed by the department’s physical therapists.

### 2.6. Statistical Analysis

All statistical computations were performed using SPSS 23.0 for Windows (IBM SPSS, Chicago, IL, USA). Statistical analyses were conducted at a 95% confidence level. Descriptive statistics characterized the population. The Wilcoxon signed-rank test evaluated the differences in the studied parameters before and after treatment. 

## 3. Results

From 2018 to 2020, seven patients that were diagnosed with a flail chest were hospitalized in our department after undergoing the ORIF procedure. The patients were aged 36–82 (mean 59.43 ± 18.88), including three females and four males. Concomitant shoulder injuries were observed in two patients, and concomitant scapula injuries in three. A total of five patients suffered shoulder mobility restrictions. The mean length of stay in the rehabilitation department was 54.00 ± 25.14 days (range 27–92). The outcomes of rehabilitation were positive in all patients. A significant reduction in pain was observed in almost all the patients (average 7.00 ± 1.83 upon admission to 4.10 ± 2.05 pre-discharge, Z = −2.07, *p* = 0.027). Delta-VAS ranged from 0 (1 patient) to 5 (mean Delta-VAS = 2.90 ± 2.02). No significant reduction in pain was observed only in one patient. Significant improvement in FIM (average of 69.43 ± 14.86 upon admission to 113.57 ± 6.40 pre-discharge, Z = −2.37, *p* = 0.018), and the Berg balance test (35.23 ± 5.87 upon admission to 49.50 ± 3.40 pre-discharge, Z = −2.37, *p* = 0.018), was observed in all patients. Upon admission, all patients required moderate to complete ADL assistance; upon discharge, all seven patients were independent in all ADL functions. ROM in the shoulder joint was limited in five patients, four patients regained full ROM, and one improved his/her ROM but retained limitations.

## 4. Discussion

Flail chest is a severe traumatic condition that can lead to complications such as acute respiratory disorders, including pneumonia, pneumothorax, prolonged ventilation of the lungs, chronic respiratory disorders, long-term pain syndrome, limitation of the shoulder girdle movement, a significant decrease in the quality of life, and often permanent disability [1]. Since the year 2000, the ORIF procedure has been actively used, significantly accelerating patients’ recovery [6,7,8,9,10,11]. However, the complication rate remains high even after undergoing the procedure. Therefore, we chose to hospitalize patients who had undergone the ORIF procedure for a flail chest in an intensive rehabilitation program according to the protocol that was developed in our department. As a result of this early and intensive rehabilitation, the pain level in the patients significantly decreased, the mobility of the shoulder girdle improved, and the functional indicators of FIM improved. We observed that the rehabilitation results were enhanced when started shortly after the operation.

This case series provides a feasibility basis for a clinical trial which would study the effectiveness of intensive rehabilitation of patients that are diagnosed with a flail chest after an ORIF procedure. We believe that the long-term effects should be estimated, including a return to work and daily activity independence. 

## 5. Conclusions

After achieving positive outcomes of early hospital-based rehabilitation of patients after an ORIF procedure, we can cautiously recommend that patients after the ORIF procedure for a flail chest be offered intensive hospital-based rehabilitation treatment. Given the small number of patients, there is a need for a full-scale clinical trial to evaluate the effect of early rehabilitation on the pain and function of patients after the surgical ORIF treatment, including the long-term consequences. Moreover, it is essential to further develop a rehabilitation protocol.

## Data Availability

All data may be presented by request to authors.
